# Prognostic impact of c-MYC and EZH2 expression in small cell and non-small cell lung carcinoma: a single-center retrospective study

**DOI:** 10.1007/s12672-026-04941-1

**Published:** 2026-04-03

**Authors:** Berkan Karadurmuş, Emel Sezer, Rabia Bozdoğan Arpacı, Kadir Eser, Semra Erdoğan, Vehbi Erçolak

**Affiliations:** 1 Division of Medical Oncology, Department of Internal Medicine, Gulhane Training & Research Hospital, General Dr. Tevfik Sağlam Street No.1, Etlik, 06010 Ankara, Türkiye; 2 Division of Oncology, Department of Internal Medicine, Medical Park Mersin Hospital, Mersin, Türkiye; 3 Department of Pathology, Faculty of Medicine, Mersin University, Mersin, Türkiye; 4 Division of Oncology, Department of Internal Medicine, Faculty of Medicine, Mersin University, Mersin, Türkiye; 5 Department of Biostatistics and Medical Informatics, Faculty of Medicine, Mersin University, Mersin, Türkiye

**Keywords:** Lung carcinoma, Non-small cell lung carcinoma, Small cell lung carcinoma, c-MYC, EZH2, Immunohistochemistry, Prognostic marker, Overall survival

## Abstract

**Background:**

c-MYC and enhancer of zeste homolog 2 (EZH2) are key regulators of cell proliferation and epigenetic reprogramming and have been implicated in the pathogenesis and progression of lung carcinoma. However, their clinicopathological and prognostic relevance in routine practice remains incompletely defined. This study aimed to evaluate the relationships between c-MYC and EZH2 immunohistochemical expression and survival in patients with small cell lung carcinoma (SCLC) and non-small cell lung carcinoma (NSCLC).

**Methods:**

This single-center retrospective study included 131 patients (98 NSCLC, 33 SCLC) followed at the outpatient clinic of medical oncology department with available archival formalin-fixed, paraffin-embedded tumor tissue. c-MYC and EZH2 expression was assessed immunohistochemically in tumor samples and evaluated by nuclear staining intensity and the percentage of stained tumor cells. For survival analyses, marker expression was dichotomized using a 30% cut-off of the percentage of stained tumor cells, and clinicopathological features were compared between groups. Overall survival (OS) was estimated using the Kaplan–Meier method and compared by the log-rank test. Prognostic factors for OS were examined using multivariate Cox proportional hazards models.

**Results:**

High c-MYC expression was associated with worse OS and an independent adverse prognostic factor in multivariate Cox regression [hazard ratio (HR) 1.86, 95% confidence interval (CI) 1.11–3.13; *p* = 0.019], together with poor performance status (ECOG ≥ 2; HR 2.91, 95% CI 1.73–4.89; *p* < 0.001). In contrast, EZH2 expression, although frequently positive and modestly correlated with c-MYC, was not significantly associated with OS.

**Conclusion:**

In this retrospective cohort of lung carcinoma patients, high c-MYC expression, but not EZH2 expression, was independently associated with poor OS after adjustment for clinical factors. c-MYC immunohistochemistry may provide additional prognostic information beyond conventional clinicopathological parameters. Larger prospective studies with standardized scoring systems are warranted to validate these findings and to clarify the clinical utility of c-MYC and EZH2 as potential prognostic and therapeutic biomarkers in lung cancer.

**Supplementary Information:**

The online version contains supplementary material available at 10.1007/s12672-026-04941-1.

## Introduction

 Lung cancer remains one of the most frequently diagnosed cancers worldwide and a leading cause of cancer-related mortality [1]. It is historically classified into small cell lung cancer (SCLC) and non-small cell lung cancer (NSCLC), the latter comprising adenocarcinoma, squamous cell carcinoma, and large cell carcinoma as the main histological subtypes [2]. Despite standard treatments and the development of targeted therapies, survival, particularly in advanced stages, remains poor, underscoring the need to identify novel prognostic factors and more effective therapeutic strategies [3]. In this setting, immunohistochemical evaluation of biologically relevant markers, such as c-MYC and EZH2, has become increasingly crucial for refining prognostic assessment and characterizing the molecular heterogeneity of lung carcinoma [4–6].

Enhancer of zeste homolog 2 (EZH2) is a key epigenetic regulator implicated in tumor cell proliferation, invasion, and migration; its overexpression in cancer cell lines has been shown to enhance proliferative, migratory, and invasive capacity, whereas EZH2 silencing by siRNA or shRNA inhibits cell growth and suppresses oncogenic potential [7]. Similarly, c-MYC is one of the most frequently activated oncogenes and encodes a transcription factor that regulates the expression of multiple genes involved in cell cycle control, growth, proliferation, apoptosis, and differentiation. Aberrant amplification and overexpression of the MYC locus occur in a substantial proportion of human malignancies and have been consistently associated with poor prognosis and, in several tumor types, with resistance to standard chemotherapeutic regimens [8]. Together, c-MYC and EZH2 influence cell proliferation, metabolism, apoptotic responses, DNA repair, and epigenetic reprogramming. Furthermore, high expression of these markers has generally been linked to adverse survival outcomes in both SCLC and NSCLC [9]. These data provide a strong biological rationale for evaluating c-MYC and EZH2 as potential prognostic biomarkers and therapeutic targets in lung carcinoma.

In SCLC, amplification of the MYC family members (c-MYC, L-MYC, and N-MYC) is frequently observed. It has been associated with a more aggressive clinical phenotype and markedly shorter survival in several series [8, 10]. High MYC activity in SCLC has also been linked to chemoresistance and to specific therapeutic vulnerabilities, including increased sensitivity to Aurora kinase inhibitors in preclinical models [11]. Additionally, in NSCLC, particularly adenocarcinoma, increased c-MYC copy number and overexpression have been reported as independent adverse prognostic factors for both disease-free survival (DFS) and overall survival (OS) [12, 13]. c-MYC promotes proliferation, invasion, metastasis, angiogenesis, and drug resistance in NSCLC, and has been shown to modulate PD-L1 expression, with high c-MYC levels correlating with increased PD-L1 expression and poorer outcomes [14]. Clinically, elevated c-MYC expression has been associated with a higher risk of recurrence even in early-stage adenocarcinoma, underscoring its potential role as a prognostic biomarker in lung cancer [12]. In NSCLC, EZH2 overexpression in clinical samples and cell lines has been associated with higher tumor stage and grade, lymph node involvement, and poorer survival, and meta-analyses have reported high EZH2 expression as an adverse prognostic factor for OS [15]. Additionally, elevated EZH2 has been linked to resistance to platinum-based chemotherapy and reduced sensitivity to tyrosine kinase inhibitors and other targeted therapies [16]. In SCLC, EZH2 is also frequently overexpressed and, in the context of RB1 and TP53 loss, contributes to increased proliferation and suppression of differentiation; although clinical data are more limited than in NSCLC, the overall trend suggests that high EZH2 expression is a marker of more aggressive tumor biology [17].

In lung cancer pathogenesis, multiple molecules and signaling pathways are involved. Data suggest c-MYC can enhance EZH2 transcription, leading to an epigenetically repressive milieu, and concomitant overexpression linked to poor prognosis [18, 19]. However, some studies show no significant survival impact of these markers. This study aimed to assess the relationship between tumoral EZH2 and c-MYC expression in small cell and non-small cell lung carcinoma, their correlation, and links to patient characteristics, overall, and disease-free survival.

## Materials and methods

### Study design and population

This single-center, retrospective observational study included patients diagnosed with lung cancer who were followed at the Medical Oncology Outpatient Clinic of Mersin University Faculty of Medicine. All consecutive patients presenting to the clinic between January 1, 2012, and April 24, 2020, with a confirmed diagnosis of lung carcinoma and available formalin-fixed, paraffin-embedded (FFPE) tumor tissue blocks in the institutional pathology archives were screened for eligibility. For all patients meeting the inclusion criteria, demographic and clinical data, Eastern Cooperative Oncology Group (ECOG) performance status [18], smoking history, histological subtype, staging information, treatment details, and OS were retrospectively collected from medical records. The relationships between these clinical parameters and the immunohistochemical expression of EZH2 and c-MYC were then analyzed. Patients were eligible if they had a follow-up diagnosis of SCLC or NSCLC, attended follow-up visits at this clinic between January 1, 2012, and April 24, 2020, were 18 years or older, and had a histopathological diagnosis of SCLC or NSCLC confirmed by the Department of Pathology at Mersin University, with sufficient archival FFPE tumor tissue available for additional immunohistochemical staining. Patients were excluded if they fell outside the predefined time frame, were younger than 18, or lacked adequate tumor tissue for further immunohistochemical analysis.

### Immunohistochemical analysis

FFPE biopsy and surgical specimens were used for immunohistochemical analysis. Tissue samples previously fixed in 10% neutral buffered formalin were processed routinely, and 5-µm-thick sections were cut from representative paraffin blocks. For immunohistochemical staining, sections were mounted on poly-L-lysine–coated slides and processed in an automated immunohistochemistry stainer (BenchMark Ultra, Ventana). Staining was performed using the ultraView Universal DAB Detection Kit (Ventana, 760 − 500) according to the manufacturer’s instructions. Scoring was performed on tumor cells only, excluding necrotic areas and non-neoplastic elements; the percentage of positively stained tumor cells was assessed in representative high-power fields.

For the detection of c-MYC expression, a rabbit monoclonal antibody against c-MYC (Cell Marque, c-MYC [EP121]) was used. EZH2 expression was assessed using a rabbit anti-human EZH2 polyclonal antibody (Leica, NCL-L-EZH2). Hematoxylin and eosin (H&E) and immunohistochemically stained sections were evaluated independently by two pathologists using an Olympus BX53 light microscope.

For both c-MYC and EZH2, nuclear staining in tumor cells was regarded as positive. Immunoreactivity was evaluated semi-quantitatively according to (i) the percentage of stained tumor cells and (ii) nuclear staining intensity. The percentage of positive tumor cells was categorized as follows: 0–10% (negative), 11–49% (low), 50–74% (medium), and 75–100% (high). Staining intensity was scored as: 0 (no staining, negative), 1+ (weak nuclear staining), 2+ (moderate nuclear staining), and 3+ (strong nuclear staining).

Based on previously published studies, an a priori cut-off value for EZH2 expression was defined as the percentage of stained tumor cells. For dichotomized analyses, ≤ 30% stained tumor cells were considered negative/low, whereas > 30% were considered positive/high. This threshold was selected to remain consistent with the literature and to standardize scoring across cases with minor modifications to the referenced scoring systems [19–21]. Importantly, this cut-off was not optimized using the present dataset or clinical outcomes.

**Fig. 1:**
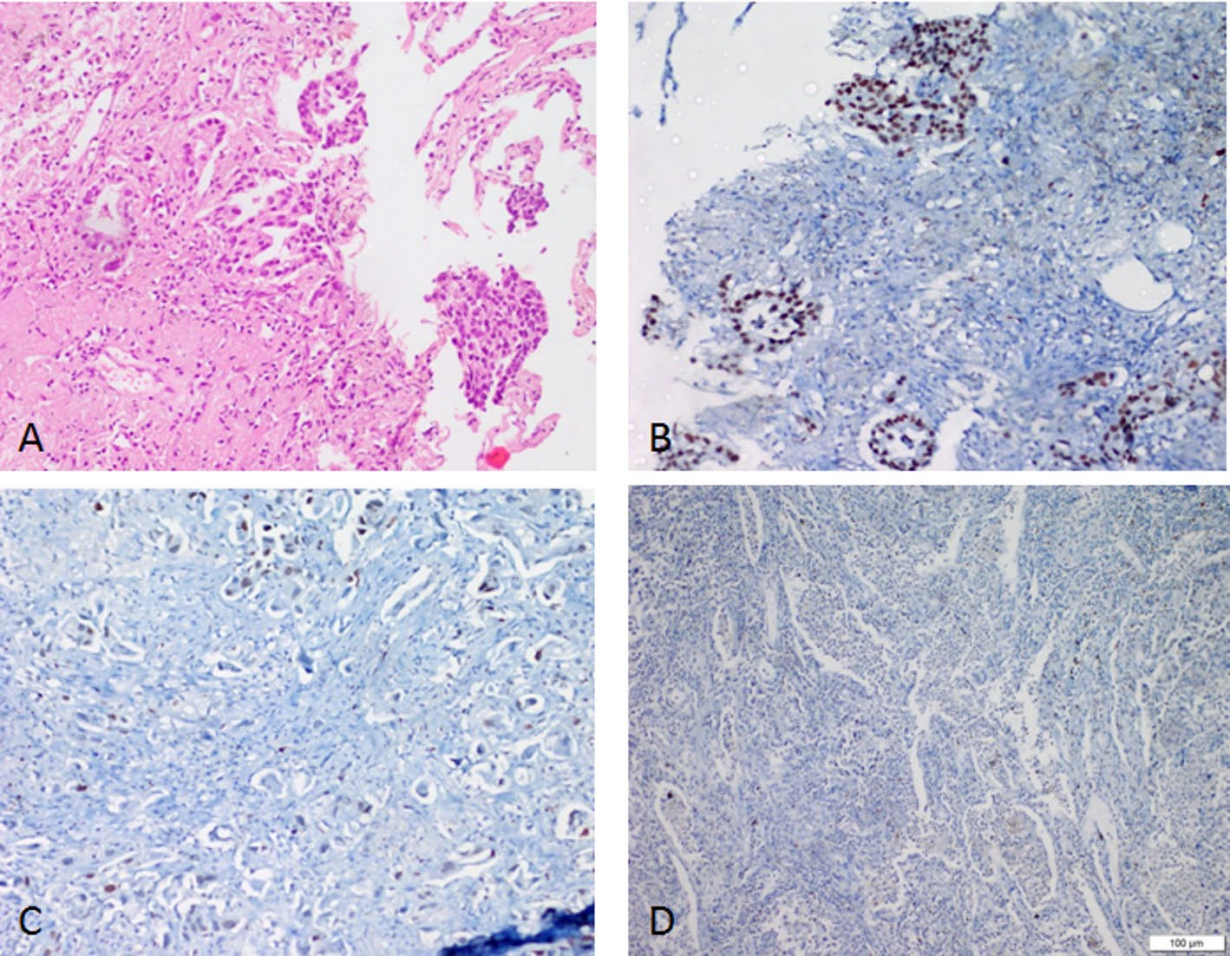
Representative immunohistochemical staining of c-MYC and EZH2 in lung carcinoma. Figure 1A illustrates the staining patterns for c-MYC, and Fig. 1B illustrates the staining patterns for EZH2. Within each part, the individual panels represent the scoring categories: (**A** hematoxylin and eosin (H&E) staining for histological orientation; **B** strong (3+) nuclear staining; **C** moderate (2+) nuclear staining; and **D** weak (1+) or negative (0) nuclear staining

Representative microphotographs of immunohistochemical staining for c-MYC and EZH2 in lung carcinoma tissues are presented in Fig. 1.

### Tumor staging

Tumor staging was performed separately for non-small cell lung cancer (NSCLC) and small cell lung cancer (SCLC). For NSCLC, clinical and pathological staging was performed according to the 8th edition of the TNM classification system of the American Joint Committee on Cancer (AJCC) [22]. Tumor (T), nodal (N), and metastatic (M) status were assigned based on available clinical, radiological, and, when applicable, histopathological findings, and patients were subsequently grouped into stage I–IV according to the AJCC 8th edition staging manual [22]. For SCLC, staging was based on the conventional two-stage system recommended by the Veterans Administration Lung Study Group (VALG), and patients were classified as having limited-stage disease (confined to one hemithorax and regional lymph nodes, within a tolerable radiotherapy field) or extensive-stage disease (tumor spread beyond the limits of limited-stage) [23]. Where applicable, TNM categories were also assigned according to the AJCC 8th edition, using the same clinical and radiological data.

### Statistical analysis

Statistical analyses were performed for all study cohorts, and survival analyses were conducted separately for patients with NSCLC and SCLC. Descriptive statistics were used to summarize patient and tumor characteristics. Categorical variables were presented as frequencies and percentages, whereas continuous variables were expressed as mean ± standard deviation (SD) or median [interquartile range, IQR], as appropriate. The distribution of continuous variables was assessed both visually and statistically, using normality tests. Group comparisons between survivors and non-survivors were performed using the chi-square test or Fisher’s exact test for categorical variables, and the independent samples t-test or Mann–Whitney U test for continuous variables, depending on distributional assumptions. OS was defined as the time from the date of diagnosis (or surgery in resected cases) to death from any cause or last follow-up. The 30% threshold for EZH2 and c-MYC was specified based on the literature and was not determined by data-driven methods. Survival curves for OS and DFS were estimated using the Kaplan–Meier method, and differences between groups (c-MYC ≤ 30% vs. > 30%) were compared using the log-rank (Mantel–Cox) test. All statistical tests were two-sided, and p-values < 0.05 were considered statistically significant. Statistical analyses were conducted using IBM SPSS Statistics for Windows, version 26.0 (IBM Corp., Armonk, NY, USA).

## Results

Baseline clinical and pathological characteristics of patients by survival status are summarized in Table 1. A total of 131 patients were included in the study. The mean age was 63.1 ± 7.8 years, and 24 patients (18.3%) were female. Most patients had good performance status, with ECOG 0–1 in 105 cases (80.2%), whereas 26 patients (19.8%) had ECOG ≥ 2. Smoking history was available for 118 patients, of whom 101 (85.6%) were smokers. Histologically, 98 patients (74.8%) had non-small cell lung cancer (NSCLC) and 33 (25.2%) had small cell lung cancer (SCLC). Among NSCLC cases, adenocarcinoma was the most common pathological subtype (61 patients, 46.6%), followed by squamous cell carcinoma (37 patients, 28.2%), while 33 patients (25.2%) had small cell carcinoma. Molecular profiling was available in 61 patients; 5 (8.2%) harbored an EGFR mutation, 1 (1.6%) had concurrent EGFR and ALK alterations, 17 (27.9%) were negative, and 38 (62.3%) had unknown molecular status. At diagnosis, metastasis was present in 38 of 98 NSCLC patients (38.8%). In the NSCLC subgroup, 60 patients (61.2%) had stage I–III disease and 38 (38.8%) had stage IV disease according to the AJCC 8th edition TNM classification. Among SCLC patients (*n* = 33), 11 (33.3%) had limited-stage and 22 (66.7%) had extensive-stage disease. Tumors were almost evenly distributed between the right (*n* = 63, 48.1%) and left lung (*n* = 68, 51.9%), most commonly involving the upper lobes (79 patients, 60.3%), followed by the lower (44 patients, 33.6%) and middle lobes (8 patients, 6.1%). Overall, 102 patients (77.9%) received systemic chemotherapy. The median overall survival for the entire cohort was 18.4 months (interquartile range, 23.5 months).

**Table 1:** Baseline characteristics of survivor and non-survivor groups

Characteristic Features	Total (*n* = 131)
Age, years	63.1 ± 7.8
Sex	
Female	24 (18.3%)
ECOG	
ECOG 0	61 (46.6%)
ECOG 1	44 (33.6%)
ECOG 2	19 (14.5%)
ECOG 3	6 (4.6%)
ECOG 4	1 (0.8%)
Smoking status (*n* = 118)	
Smoker	101 (85.6%)
Histological type	
Small cell lung carcinoma (SCLC)	33 (25.2%)
Non-small cell lung carcinoma (NSCLC)	98 (74.8%)
Pathological diagnosis	
Adenocarcinoma	61 (46.6%)
Squamous cell carcinoma	37 (28.2%)
Small cell carcinoma	33 (25.2%)
Molecular profiling (*n* = 61)	
EGFR mutation	5 (8.2%)
EGFR + ALK mutation	1 (1.6%)
Negative	17 (27.9%)
Unknown	38 (62.3%)
Metastasis at diagnosis (NSCLC only) (*n* = 98)	38 (38.8%)
Staging of NSCLC (*n* = 98)	
Stage I-III	60 (61.2)
Stage IV	38 (38.8%)
Staging of SCLC (*n* = 33)	
Limited stage	11 (33.3%)
Extensive stage	22 (66.7%)
Tumor laterality	
Right lung	63 (48.1%)
Left lung	68 (51.9%)
Lobar localization	
Upper lobe	79 (60.3%)
Lower lobe	44 (33.6%)
Middle lobe	8 (6.1%)
Chemotherapy received	102 (77.9%)
Overall Survival, months	18.4 [23.5]

Values are presented as numbers (%), mean ±standard deviation, or median [interquartile range]

Spearman correlation analysis demonstrated strong internal consistency among the different EZH2 scoring components (Table 2). The global EZH2 score showed strong positive correlations with the semi-quantitative tumor staining category, the percentage of stained tumor cells, and the staining intensity (rho = 0.65–0.78, *p* < 0.001), and these EZH2-derived variables were also highly correlated with one another (rho up to 0.96, *p* < 0.001). Similarly, very strong positive correlations were observed among all c-MYC variables: the overall c-MYC score, tumor staining category, percentage of stained tumor cells, and staining intensity were closely interrelated (rho = 0.82–0.96, all *p* < 0.001). Cross-marker analysis revealed weak-to-modest, but generally significant, positive correlations between EZH2 and c-MYC expression. Higher EZH2 tumor staining and percentage of stained cells were associated with higher c-MYC tumor staining, percentage, and staining intensity (rho approximately 0.18–0.25, *p* ≤ 0.05 for most pairs).

**Table 2:** Spearman correlation coefficients between EZH2 and c-MYC immunohistochemical variables (*n* = 131)

	EZH2	EZH2 tumor stainingᵃ	EZH2% stained cells	EZH2 staining intensity	c-MYC	c-MYC tumor stainingᵃ	c-MYC % stained cells	c-MYC staining intensity
EZH2	1.000	0.771**	0.777**	0.647**	0.163	0.216*	0.224*	0.181*
EZH2 tumor stainingᵃ		1.000	0.955**	0.731**	0.188*	0.252**	0.243**	0.237**
EZH2% stained cells			1.000	0.732**	0.179*	0.244**	0.250**	0.248**
EZH2 staining intensity				1.000	0.141	0.158	0.160	0.184*
c-MYC					1.000	0.868**	0.889**	0.824**
c-MYC tumor stainingᵃ						1.000	0.961**	0.899**
c-MYC % stained cells							1.000	0.871**
c-MYC staining intensity								1.000

*Correlation is significant at the 0.01 level (2-tailed)

**Correlation is significant at the 0.05 level (2-tailed)

ᵃOrdinal semi-quantitative tumor staining category

Table 3 shows the baseline clinicopathological characteristics stratified by EZH2 and c-MYC expression levels. When patients were stratified according to EZH2 expression (≤ 30% vs. > 30% stained tumor cells), there were no significant differences in age, ECOG performance status, histological subtype, tumor stage, tumor laterality, lobar localization, or receipt of chemotherapy (all *p* > 0.05). However, female sex was more frequent in the EZH2-low group (31.4% vs. 13.5%, *p* = 0.019), whereas current or former smoking was more common among patients with EZH2 expression > 30% (89.7% vs. 74.2%, *p* = 0.035). The proportion of metastatic disease at diagnosis among NSCLC patients was numerically higher in the EZH2-low group, but this did not reach statistical significance (52.2% vs. 34.7%, *p* = 0.13). In contrast, c-MYC expression levels were clearly associated with tumor histology. Age, sex distribution, ECOG status, smoking history, stage, tumor laterality, lobar localization, and use of chemotherapy were similar between patients with c-MYC ≤ 30% and > 30% (all *p* > 0.05). High c-MYC expression (> 30%) was significantly associated with NSCLC histology (*p* < 0.001), and with pathological subtype (*p* < 0.001), with a higher proportion of squamous cell carcinoma and a lower proportion of small cell carcinoma in the c-MYC-high group, whereas the c-MYC-low group contained relatively more small cell carcinoma. Metastatic disease at diagnosis was more common among patients with c-MYC > 30%, but the difference did not reach statistical significance (44.6% vs. 27.3%, *p* = 0.096).

**Table 3:** Baseline clinicopathological characteristics stratified by EZH2 and c-MYC expression levels

	EZH2 ≤ 30% (*n* = 35)	EZH2 > 30% (*n* = 96)	*p*	c-myc ≤ 30% (*n* = 61)	c-myc > 30% (*n* = 70)	*p*
Age, years	63.7 ± 8.2	63.2 ± 7.6	0.73	63.6 ± 7.3	63.1 ± 8.2	0.72
Sex						
Female	11 (31.4)	13 (13.5)	0.019	14 (23.0)	10 (14.3)	0.20
ECOG			0.89			0.49
ECOG 0	17 (48.6)	44 (45.8)		31 (50.8)	30 (42.9)	
ECOG 1	10 (28.6)	34 (35.4)		17 (27.9)	27 (38.6)	
ECOG 2	6 (17.1)	13 (13.5)		10 (16.4)	9 (12.9)	
ECOG 3	2 (5.7)	4 (4.2)		2 (3.3)	4 (5.7)	
ECOG 4	-	1 (1.0)		1 (1.5)	-	
Smoking status						
Smoker	23 (74.2)	78 (89.7)	0.035	46 (85.2)	55 (85.9)	1.0
Histological type						
SCLC	23 (65.7)	75 (78.1)	0.15	33 (54.1)	65 (92.9)	< 0.001
NSCLC	12 (34.3)	21 (21.9)		28 (45.9)	5 (7.1)	
Pathological diagnosis						
Adenocarcinoma	18 (51.4)	43 (44.8)	0.078	28 (45.9)	33(47.1)	< 0.001
Squamous cell carcinoma	5 (14.3)	32 (33.3)		5 (8.2)	32 (45.7)	
Small cell carcinoma	12 (34.3)	21 (21.9)		28 (45.9)	5 (7.1)	
Molecular profiling (*n* = 61)						
EGFR mutation	3 (16.7)	2 (4.6)	0.42	2 (7.1)	3 (9.1)	0.81
EGFR + ALK mutation	-	1 (2.3)		-	1 (3.0)	
Negative	5 (27.7)	12 (27.9)		8 (28.5)	9 (27.3)	
Unknown	10 (55.6)	28 (65.2)		18 (64.4)	20 (60.6)	
Metastasis at diagnosis (NSCLC only)	12 (52.2)	26 (34.7)	0.13	9 (27.3)	29 (44.6)	0.096
Staging of NSCLC (*n* = 98)						
Stage I-III	11 (47.8)	49 (65.3)	0.13	24 (72.7)	36 (55.4)	0.096
Stage IV	12 (52.2)	26 (34.7)		9 (27.3)	29 (44.6)	
Staging of SCLC (*n* = 33)						
Limited stage	5 (41.7)	6 (28.6)	0.44	8 (28.6)	3 (60.0)	0.30
Extensive stage	7 (58.3)	15 (71.4)		20 (71.4)	2 (40.0)	
Tumor laterality						
Right lung	14 (40.0)	49 (51.0)	0.26	27 (44.3)	36 (51.4)	0.41
Left lung	21 (60.0)	47 (49.0)		34 (55.7)	34 (48.6)	
Lobar localization						
Upper lobe	21 (60.0)	58 (60.4)	0.17	36 (59.0)	43 (61.4)	0.78
Lower lobe	14 (40.0)	30 (31.3)		22 (36.1)	22 (31.4)	
Middle lobe	-	8 (8.3)		3 (4.9)	5 (7.1)	
Chemotherapy received	28 (80.0)	74 (77.1)	0.72	48 (78.7)	54 (77.1)	0.83
Overall Survival	19.8 [23.6]	17.8 [22.2]	0.92	20.4 [26.9]	14.6 [23.1]	0.24

Values are presented as numbers (%), mean ±standard deviation, or median [interquartile range]

**Fig. 2:**
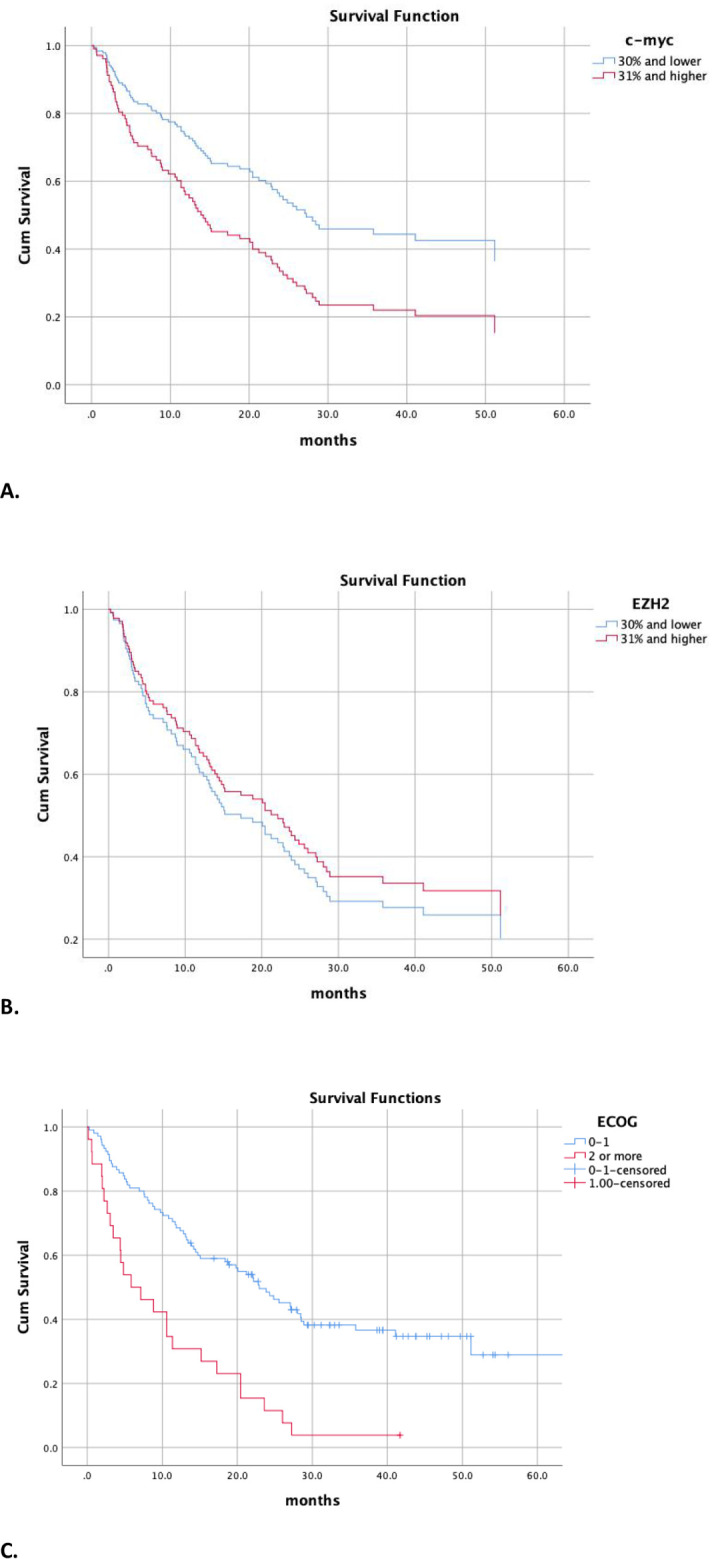
Kaplan–Meier curves for overall survival according to c-MYC and EZH2 expression: **A** Overall survival stratified by c-MYC expression, dichotomized using a 30% cut-off for the percentage of stained tumor cells (≤ 30% vs. > 30%) (log-rank *p* < 0.001). **B** Overall survival stratified by EZH2 expression, dichotomized using the same 30% cut-off (≤ 30% vs. > 30%) (log-rank *p* = 0.314) **C** Overall survival stratified by performance status (log-rank *p* < 0.001)

In the multivariate Cox proportional hazards model 1, including age, sex, smoking status, ECOG performance status, tumor type (NSCLC vs. SCLC), and EZH2 expression, only performance status was independently associated with overall survival. Poor performance status (ECOG ≥ 2) was a strong adverse prognostic factor (HR = 2.86, 95% CI: 1.69–4.83, *p* < 0.001), indicating approximately a 2.9-fold increased risk of death compared with patients with ECOG 0–1. Age, sex, and smoking status were not significantly associated with overall survival (all p-values > 0.40). Likewise, tumor type (NSCLC vs. SCLC) (HR = 1.09, 95% CI: 0.64–1.85, *p* = 0.742) and dichotomized EZH2 expression (≤ 30% vs. > 30%) (HR = 0.85, 95% CI: 0.51–1.41, *p* = 0.526) showed no independent prognostic impact in this model. In the multivariate Cox proportional hazards model 2, incorporating age, sex, smoking status, ECOG performance status, tumor type, and c-MYC expression, ECOG performance status and c-MYC remained independently associated with overall survival. Poor performance status (ECOG ≥ 2) was a strong adverse prognostic factor (HR = 2.91, 95% CI: 1.73–4.89, *p* < 0.001), indicating approximately a threefold increased risk of death compared with patients with ECOG 0–1. High c-MYC expression (> 30% stained tumor cells) was also independently associated with worse overall survival (HR = 1.86, 95% CI: 1.11–3.13, *p* = 0.019). Age, sex, smoking status, and tumor type (NSCLC vs. SCLC) were not significantly related to overall survival in this model (all *p* > 0.10). To account for potential heterogeneity between histologic subtypes, we repeated the multivariable analysis in the NSCLC subgroup. In this NSCLC-only model, c-MYC positivity remained an independent prognostic factor (HR 1.979, 95% CI 1.070–3.660, *p* = 0.029). In a separate NSCLC-only model including EZH2, EZH2 was not independently associated with prognosis (HR 0.712, 95% CI 0.384–1.319, *p* = 0.280) (Supplementary Table). Kaplan-Meier curves for OS by c-MYC and EZH2 expression (30% cut-off) are shown in Fig. 2A–B. In Fig. 2C, Kaplan-Meier curves by performance status (ECOG) are shown (Table 4).

**Table 4:** Multivariate Cox proportional hazards models for overall survival

	Model 1 (with EZH2) HR (95% CI)	*p*	Model 2 (with c-MYC) HR (95% CI)	*p*
Age, years	0.998 (0.966–1.031)	0.902	1.002 (0.970–1.035)	0.905
Sex (female vs. male)	1.289 (0.658–2.523)	0.460	1.223 (0.616–2.430)	0.565
Smoking (yes vs. no)	0.924 (0.482–1.772)	0.812	0.982 (0.511–1.886)	0.955
ECOG performance status (≥ 2 vs. 0–1)	2.855 (1.690–4.825)	< 0.001	2.909 (1.730–4.891)	< 0.001
Tumor type (NSCLC vs. SCLC)	1.093 (0.644–1.853)	0.742	1.545 (0.859–2.781)	0.147
EZH2 (> 30% vs. ≤ 30% stained tumor cells)	0.849 (0.512–1.408)	0.526	–	–
c-MYC (> 30% vs. ≤ 30% stained tumor cells)	–	–	1.863 (1.110–3.129)	0.019

HR: hazard ratio; CI: confidence interval. Reference categories: male sex, non-smoker, ECOG 0–1, SCLC, EZH2 ≤ 30%, c-MYC ≤ 30%

## Discussion

This retrospective study evaluated the clinicopathological correlation and prognostic significance of c-MYC and EZH2 immunohistochemical expression in patients with SCLC and NSCLC followed at a single tertiary center. The main findings showed that high c-MYC expression, dichotomized at a 30% cutoff of the percentage of stained tumor cells, was independently associated with worse OS; however, EZH2 expression, although frequently detectable, was not significantly associated with OS. Our data are consistent with the growing evidence that c-MYC overexpression is associated with more aggressive disease biology in lung cancer. In the present cohort, patients with c-MYC expression > 30% had a shorter median OS than those with lower expression, and this effect persisted after adjustment for performance status and tumor type.

The high c-MYC expression indicates a clinically significant increase in the risk of death, independent of established clinical prognostic factors. These findings align with previous reports suggesting that c-MYC activation is associated with poorer outcomes in NSCLC. Similarly, a study of 128 patients with surgically resected primary NSCLC showed that the c-MYC group had lower OS, and the risk was higher in patients with concurrent PD-L1 and c-MYC expression [14]. Moreover, a study on 104 patients with surgically resected SCLC stated that c-MYC expression is a risk factor for shorter OS regardless of sex, COPD, and adjuvant treatment [24]. In addition, another study of 77 patients with SLCL found that patients with MYC amplification had significantly shorter survival than those without MYC amplification [25]. All in all, our results support the conclusion that c-MYC is an adverse prognostic marker in lung carcinoma and may help refine risk stratification beyond conventional clinicopathological factors.

The lack of prognostic impact of EZH2 expression in our series contrasts with some studies reporting an association between EZH2 overexpression and poor survival in lung carcinoma. In NSCLC, a meta-analysis found that high expression of EZH2 indicates poor prognosis, possibly related to tumor stage and cancer type [7]. On the other hand, in a study of 40 surgically resected SCLC patients, no significant association was found between EZH2 expression levels and clinicopathological characteristics or postoperative survival, consistent with our observation that EZH2 expression did not exert a clear prognostic impact in our cohort [26]. Several explanations may account for this discrepancy. Compared with Behrens et al., who evaluated predominantly surgically resected NSCLC [4], our cohort was more clinically heterogeneous, which may attenuate EZH2’s subtype- and treatment-dependent prognostic signals. Similarly, while Fan et al. found that high EZH2 levels generally indicate a worse prognosis in NSCLC, their meta-analysis also revealed significant variability across studies, suggesting that the effect may vary by stage distribution, histologic type, and treatment context [7]. Methodological non-equivalence is a key issue: EZH2 assessment varies across antibodies/platforms, and “high EZH2” is defined differently, affecting group assignment. Therefore, the discrepancy between our negative findings and prior positive reports may reflect both cohort composition and scoring differences rather than a uniform absence of prognostic relevance for EZH2.

The weak-to-moderate correlations observed between EZH2 and c-MYC expression are biologically plausible, given that experimental studies have suggested functional links between MYC signaling and EZH2-mediated epigenetic repression [27, 28]. In our material, higher EZH2 tumor staining and percentage of stained cells were modestly associated with higher c-MYC expression, indicating that co-activation of these two markers occurs in a subset of tumors. However, the correlation coefficients were low enough to suggest only partial overlap between these pathways in routine clinical specimens. Clinically, this pattern may reflect the heterogeneity of oncogenic programs in lung cancer, where multiple parallel or converging pathways contribute to tumor behavior. Combining c-MYC and EZH2 status did not provide additional prognostic information beyond c-MYC alone in our dataset; however, the biological interplay between these markers may still be relevant to the design of future targeted or combination therapies. Preclinical and translational data support functional crosstalk between MYC signaling and EZH2-mediated chromatin regulation, including evidence in lung cancer models [28, 29]. It represents a biologically distinct, potentially more aggressive “double-hit” subgroup characterized by simultaneous proliferative drive and epigenetic repression. Although we were not powered to robustly test this hypothesis, co-expressing cases could theoretically be enriched for epigenetic vulnerabilities and may warrant evaluation in larger, histology-stratified cohorts and treatment-focused studies.

Performance status, as expected, emerged as the strongest independent predictor of OS in our cohort, consistent with large series showing ECOG PS as one of the most powerful prognostic factors in both advanced NSCLC and SCLC [30, 31]. According to our findings, patients with ECOG ≥ 2 had nearly a threefold increased risk of death compared with those with ECOG 0–1. In contrast, age, sex, and smoking history were not independently associated with survival, which may reflect the relatively homogeneous, predominantly older, and heavily smoking population typical of a real-life lung cancer clinic, in line with previous real-world studies [32]. Tumor type (NSCLC vs. SCLC) also did not retain independent significance in the multivariate analyses, probably because the adverse effect of SCLC histology is partly captured by performance status, disease burden, and treatment patterns, and because the number of SCLC cases was limited.

This study has limitations, including its retrospective, single-center, small-cohort design, which introduces selection and information bias, particularly regarding data accuracy. The study relied on immunohistochemistry as a proxy for genetic or epigenetic changes, without molecular profiling for MYC, chromosomal rearrangements, or EZH2 mutations. Additionally, the cutoffs for c-MYC and EZH2, while supported by internal analyses and the literature, underscore the lack of standardization in marker scoring. Variability in treatment over time may also influence outcomes. Despite these limitations, it has strengths: it reflects real-world data on NSCLC and SCLC, provides systematic clinicopathological and survival information, and includes detailed protein-level analysis. High c-MYC expression consistently links to poor OS, highlighting its prognostic potential, whereas EZH2’s role remains unclear and warrants further standardized research.

In conclusion, our findings indicate that c-MYC, but not EZH2, is an independent adverse prognostic factor in lung carcinoma, after adjustment for performance status and other clinical variables. c-MYC immunohistochemistry may help refine risk stratification beyond conventional clinicopathological parameters, particularly in NSCLC. Future prospective, larger-scale studies integrating immunohistochemistry with molecular analyses are warranted to validate these results, to better define standardized scoring systems and cut-off values for c-MYC and EZH2, and to explore whether these markers can guide targeted or combination therapeutic strategies in lung cancer.

## Electronic Supplementary Material

Below is the link to the electronic supplementary material.

Supplementary Material 1.

## Data Availability

The data that support the findings of this study are available from the corresponding author upon reasonable request.
