# Supplementary material for: Prognostic impact of c-MYC and EZH2 expression in small cell and non-small cell lung carcinoma: a single-center retrospective study
Source: Discov Oncol. 2026 Apr 3;17:730. doi: 10.1007/s12672-026-04941-1 (PMC13172210; doi:10.1007/s12672-026-04941-1)
Supplement: Supplementary file 1 — Supplementary Material 1. [file 12672_2026_4941_MOESM1_ESM.docx]

**Supplementary Table: Multivariable Cox regression analysis restricted to NSCLC patients**

| **Model 1** | **HR** | **95% CI** | **p-value** |
| --- | --- | --- | --- |
| **Sex** | 0.946 | 0.405–2.206 | 0.897 |
| **Age** | 1.003 | 0.968–1.039 | 0.871 |
| **Smoking** | 0.902 | 0.422–1.931 | 0.791 |
| **c-MYC** | 1.979 | 1.070–3.660 | 0.029 |
| **ECOG category** | 3.404 | 1.861–6.229 | < 0.001 |
| **Model 2** | **HR** | **95% CI** | **p-value** |
| **Sex** | 1.117 | 0.477–2.614 | 0.799 |
| **Age** | 0.996 | 0.961–1.033 | 0.836 |
| **Smoking** | 0.796 | 0.374–1.692 | 0.553 |
| **ECOG category** | 3.111 | 1.645–5.882 | < 0.001 |
| **EZH2** | 0.712 | 0.384–1.319 | 0.280 |
